# Non-Invasive *Drosophila* ECG Recording by Using Eutectic Gallium-Indium Alloy Electrode: A Feasible Tool for Future Research on the Molecular Mechanisms Involved in Cardiac Arrhythmia

**DOI:** 10.1371/journal.pone.0104543

**Published:** 2014-09-16

**Authors:** Po-Hung Kuo, Te-Hsuen Tzeng, Yi-Chun Huang, Yu-Hao Chen, Yi-Chung Chang, Yi-Lwun Ho, June-Tai Wu, Hsiu-Hsian Lee, Po-Jung Lai, Kwei-Yan Liu, Ya-Chen Cheng, Shey-Shi Lu

**Affiliations:** 1 Graduate Institute of Electronics Engineering, National Taiwan University, Taipei, Taiwan; 2 Graduate Institute of Biomedical Electronics and Informatics, National Taiwan University, Taipei, Taiwan; 3 Center for Dynamical Biomarkers and Translation Medicine, National Central University, Taoyuan, Taiwan; and Research Center for Adaptive Data Analysis, National Central University, Taoyuan, Taiwan; 4 Section of Cardiology, Department of Internal Medicine, National Taiwan University Hospital and National Taiwan University College of Medicine, Taipei, Taiwan; 5 Institute of Molecular Medicine, College of Medicine, National Taiwan University, Taipei, Taiwan; CNR, Italy

## Abstract

**Background:**

*Drosophila* heart tube is a feasible model for cardiac physiological research. However, obtaining *Drosophila* electrocardiograms (ECGs) is difficult, due to the weak signals and limited contact area to apply electrodes. This paper presents a non-invasive Gallium-Indium (GaIn) based recording system for *Drosophila* ECG measurement, providing the heart rate and heartbeat features to be observed. This novel, high-signal-quality system prolongs the recording time of insect ECGs, and provides a feasible platform for research on the molecular mechanisms involved in cardiovascular diseases.

**Methods:**

In this study, two types of electrode, tungsten needle probes and GaIn electrodes, were used respectively to noiselessly conduct invasive and noninvasive ECG recordings of *Drosophila*. To further analyze electrode properties, circuit models were established and simulated. By using electromagnetic shielded heart signal acquiring system, consisted of analog amplification and digital filtering, the ECG signals of three phenotypes that have different heart functions were recorded without dissection.

**Results and Discussion:**

The ECG waveforms of different phenotypes of *Drosophila* recorded invasively and repeatedly with n value (n>5) performed obvious difference in heart rate. In long period ECG recordings, non-invasive method implemented by GaIn electrodes acts relatively stable in both amplitude and period. To analyze GaIn electrode, the correctness of GaIn electrode model established by this paper was validated, presenting accuracy, stability, and reliability.

**Conclusions:**

Noninvasive ECG recording by GaIn electrodes was presented for recording *Drosophila* pupae ECG signals within a limited contact area and signal strength. Thus, the observation of ECG changes in normal and SERCA-depleted *Drosophila* over an extended period is feasible. This method prolongs insect survival time while conserving major ECG features, and provides a platform for electrophysiological signal research on the molecular mechanism involved in cardiac arrhythmia, as well as research related to drug screening and development.

## Introduction

Recent studies have investigated the electrocardiographs (ECGs) of certain insects for modeling and understanding human-like hearts. Cardiac morphology has evolved from the simple tubular structure observed in arthropods [1] to the multi chambered structure observed in mammals. The heart tube of *Drosophila* is one of the most primitive heart systems in the animal kingdom. The propagation of action potential, contraction of cardiomyocytes, and transcriptional program specifying heart development were conserved from *Drosophila* to humans; thus, the *Drosophila* heart tube is a feasible model for conducting cardiac physiological research. However, obtaining *Drosophila* ECGs is difficult, because the *Drosophila* heart emits weak signals and offers a limited contact area to apply electrodes [2]. Current techniques for conducting *Drosophila* ECG measurements include (1) using sharpened tungsten electrodes, which are inserted laterally into the abdomen during the early pupal stage of the animal [3], and (2) using a suction electrode to obtain ECGs during the larval stage of the animal, which is pulled outside the animal and bathed in a physiological solution [4]. All of these methods are invasive, and the ECG recording time involved in using these methods is limited by the survival time of *Drosophila*. Moreover, heart-brain cross-talk may be damaged by the changes in autonomic tone induced by invasive procedures [5]. For measurements over extended periods of time, such as over ten minutes, a noninvasive method must be used to prolong the survival time of the animal. To develop a noninvasive ECG measurement method, this study investigated the application of a novel liquid metal, Gallium-Indium (GaIn), as a material used in recording electrodes.

GaIn is a liquid eutectic alloy with a low melting point and low impedance, and is thus a feasible material for use in noninvasive ECG electrodes [2] [6]. Liquid GaIn enables the electrode interface to make tight contact with the *Drosophila* skin, thereby lowering noise and improving the signal quality without necessitating the destruction of the animal's body. This noninvasive system provides a useful platform for continually conducting *Drosophila* ECG measurements.

This paper presents a recording system for ECG measurement, permitting the heart rate and heartbeat features of *Drosophila* to be observed. This novel, high-signal-quality, GaIn-based, noninvasive electrode interface system enables the recording time of insect ECGs to be prolonged, and is a feasible tool for the future development of a platform for research on the molecular mechanisms involved in cardiac arrhythmia.

## Materials and Methods

### Electrodes

In this study, two types of electrode, tungsten needle probes and GaIn electrodes were respectively used to conduct invasive and noninvasive ECG recordings of *Drosophila* specimens. The tungsten needle probes, which had a length of 76 mm, a diameter of 257 µm, and 0.3 mm exposed tips, were coated with biocompatible parylene-C for noise insulation, and were fabricated by A-M Systems.

With melting point of 15.7°C, GaIn is a liquid metal with low interface impedance at room temperature, thus causing low noise and baseline wander, which is essential for recording weak signals [7]. GaIn was carried by tungsten probes to create contact electrodes with limited signal attenuation and distortion for noninvasive recording; using this method, the recorded signal strength of the *Drosophila* cardiac signal was on the order of nanovolts before amplification.

### Modeling, simulation, and impedance analysis

To investigate the electrical performance of the various ECG electrodes used in this study, particularly the impedance and recorded ECG waveforms, circuit models of the three different electrodes were established, and their ECG recording performance was simulated using MATLAB software (MathWorks, Inc.).

**Figure 1:**
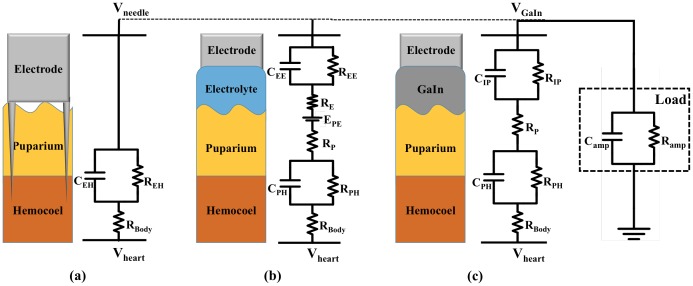
Pupa-electrode interface and its electrical equivalent model circuit of (a) invasive tungsten needle probe electrode (b) non-invasive jelled electrode (c) GaIn liquid metal electrode.

Following [8], a circuit model of an invasive electrode was generated, as shown in Figure 1(a), in which C_EH_ and R_EH_ are the impedances in parallel at the interface between the tungsten needle probe and the hemocoel; R_Body_ is the DC resistance of the hemocoel. The equivalent circuit of the amplifier load connects to the tungsten needle probe and measures the heart signal, expressed as V_needle_ For gelled electrodes, as shown in Figure 1(b), R_E_ is the electrolyte DC resistance and R_P_ is the puparium DC resistance, C_EE_//R_EE_ is the interface impedance between the electrode and the electrolyte, and C_PH_//R_PH_ is the interface impedance between the puparium and the hemocoel [9]. In addition to the interface impedance and DC resistance, the fact that the puparium is an ion-semipermeable membrane must be considered: E_PE_ is the potential difference caused by differences in ionic concentration across this membrane, and is obtained using the Nernst equation [10].

Using wet electrodes, such as electrolyte electrodes, to record *Drosophila* ECGs noninvasively, demonstrates the disadvantage of exhibiting motion artifacts caused by the electrolyte gel-skin potential, expressed as E_PE_. To avoid ionization and ion diffusion of the electrode-skin interface, the GaIn liquid metal that is similar to a dry electrode is used for replacing electrolytes to measure *Drosophila* heart signals, expressed as V_GaIn_, which is monitored without an ionic potential difference. Therefore, only interface impedance and DC resistance must be considered, as shown in Figure 1(c). The equations for these model circuits and simulated results are discussed in the following section.

### Drosophila strains, rearing, and crosses

To deplete intracellular Ca^2+^ levels, we performed tissue-specific knockdown of the expression of sarcoendoplasmic reticulum Ca^2+^-ATPase (SERCA) [11] by using the GAL4-UAS bipartite expression system [12]. A heart-specific GAL4 line, *NP1029-GAL4* (expressed fluorescent protein compared with wild-type *Drosophila*) [13], was crossed with *UAS-SERCA-dsRNA* (Bloomington stock number 25928). The F1 progeny, *NP1029-GAL4*/+;*UAS-SERCA-dsRNA*/+ (SERCA-depleted), exhibited expression of the GAL4 transcription factor in the heart, but not in other muscle tissues, to drive the expression of *SERCA-dsRNA,* resulting in the RNAi-mediated degradation of *SERCA mRNA*, reduced expression of SERCA, and depletion of sarcoendoplasmic reticulum calcium ions in cardiomyocytes. *NP1029-GAL4*/+;*UAS-mCD8-GFP*/+ (NP) was used as the control. Both healthy and SERCA-depleted *Drosophila* were raised on a standard sucrose-agar fly medium at 25°C.

**Figure 2:**
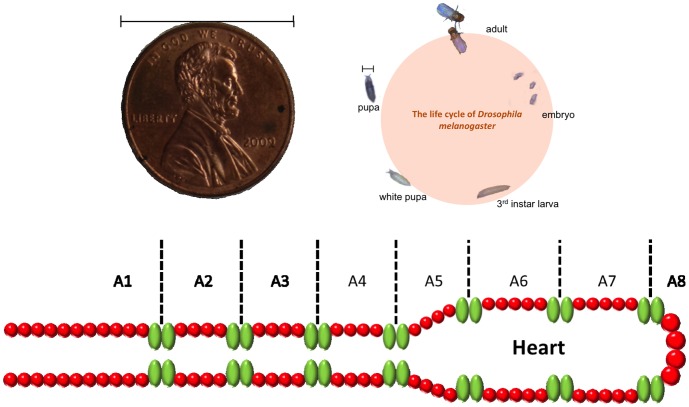
*Drosophila* life cycle and schematic illustration of the pupa heart tube structure. In which the size of *Drosophila* is very tiny compared with the coin, which limited the contact area of electrodes, causing the difficulty of measurement.

Canton-S (CS, wild type *Drosophila*) and *UAS-SERCA-dsRNA Drosophila* specimens were obtained from the Bloomington *Drosophila* Stock Center (Bloomington, IN). *NP1029-GAL4* was obtained from the Kyoto Stock Center at the *Drosophila* Genetic Resource Center. Flies were raised on a standard sucrose-agar fly medium at 25°C. We set up a genetic cross to produce SERCA-depleted, in which SERCA expression in the heart was depleted. CS and NP were used as the controls. To study heart function, we collected white pupae which were immobile and semitransparent for experimentation, as shown in Figure 2. At this stage, the heartbeat could be easily observed and recorded because of the transparency and immobility of the specimens; thus, dissection and anesthesia were unnecessary.

### Electrophysiological experiment

**Figure 3:**
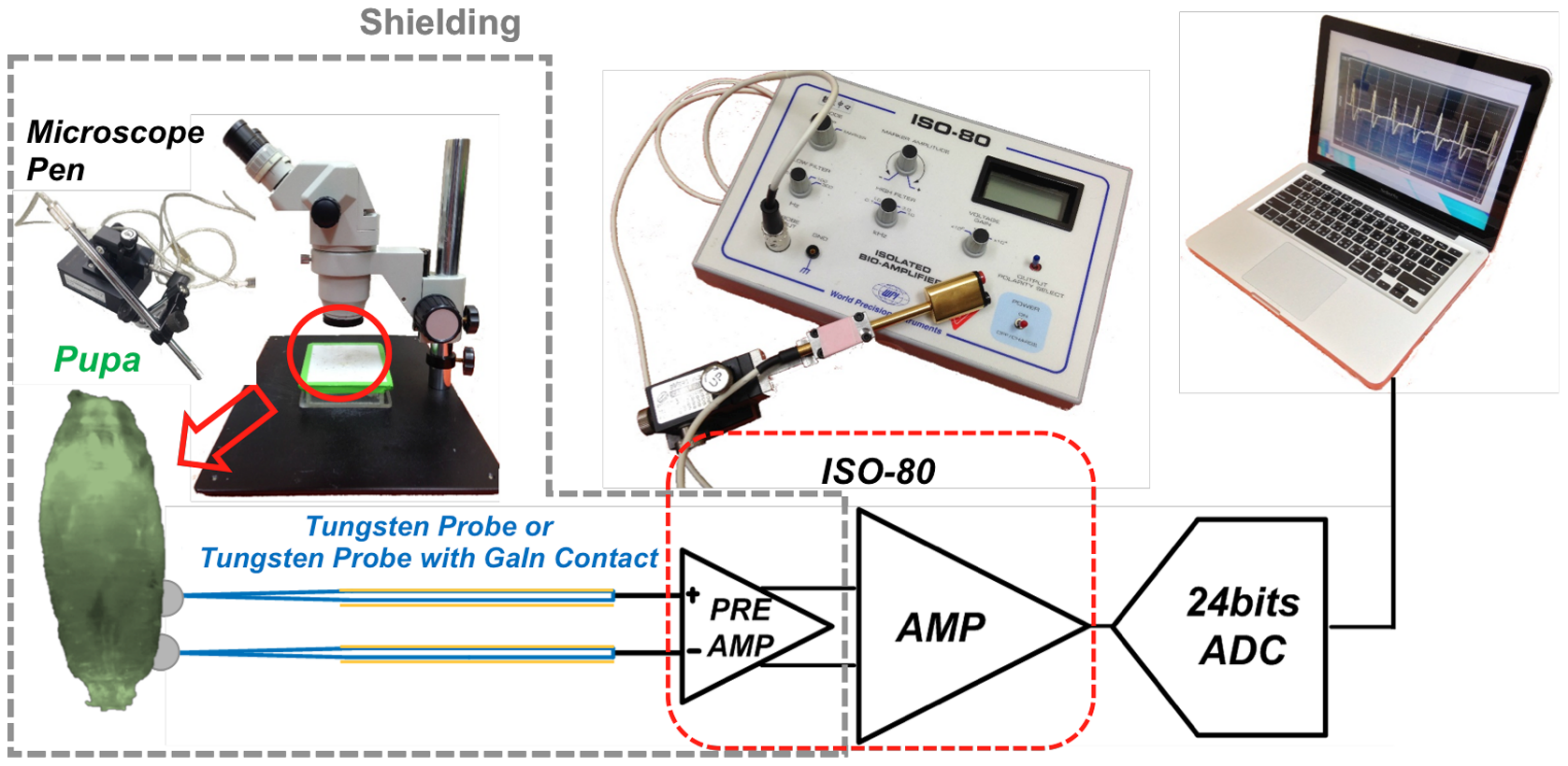
System of electrophysiological experiment.

The heart rates and ECG data of the *Drosophila* specimens were recorded to analyze their cardiac physiology. For conducting distinct dissected heart recording [14], in this study, the heart performance of the pupae were measured directly by using invasive and non-invasive method without dissection. Avoiding dissection in conducting measurements enables the physiological destruction of the animal to be avoided, thereby preventing electrical errors and enabling ECGs to be recorded over extended periods. In this study, by using the channel-blocked method, we recorded the ECGs of three phenotypes of *Drosophila*: CS, NP1029, and SERCA-depleted. Figure 3 shows the setup of the electrophysiological experiment. The heart signals of *Drosophila* are weak and particularly difficult to distinguish by noninvasive methods. Averting electromagnetic (EM) noise during experiments is thus crucial. To reduce noise, the experiment was conducted in a shielding box. To avoid interference caused by muscle potential, white pupae were used for static recording. In the invasive method, two tungsten probes (A-M Systems) were inserted at A6–A8 under the skin of each pupa to record the ECG; this process was monitored using a microscope. To ensure that probes were precisely inserted in the correct location, a three-axis microscope pen was used to perform Z-axis observation; this pen defined the precise Z-location of the recording probes. In the noninvasive method, GaIn carried by tungsten probes was placed in contact with the surface of the animals at A6–A8.

After the electrodes were placed, the two tungsten probes were connected to a two-stage cascaded amplifier (ISO-80, World Precision). The amplifier consisted of a pre-amplifier and amplifier, and provided low noise AC coupled amplification with maximum gain of 80 dB. In which, the lowest low-pass setting is 5 Hz and the upper passband is 10 kHz. Following amplification, an analog-to-digital converter (ADC) was used to digitize the analog signals; the ADC sampling rate was 2.6 KHz, with a resolution of 24 bits. After that, the digitized signals were formatted to USB, and sent to a laptop via a USB port, to be displayed as waveforms. Because of the weakness of the signals, all equipment was powered by battery to obtain a high signal-to-noise ratio.

**Figure 4:**
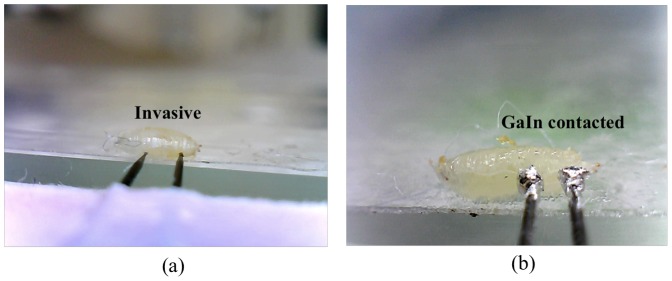
Photos of (a) invasive and (b) GaIn based non-invasive ECG recording.

Figure 4 presents the *Drosophila* ECGs. Images recorded using the invasive and noninvasive methods are shown in Figures 4(a) and 4(b), respectively. In the noninvasive method, as shown in Figure 4(b), the tungsten electrodes were coated with parylene-C, an insulator against noise.

### Signal Processing

The sampling frequency of the ECG signal was 2604 Hz. Each signal was preprocessed using a second-order infinite impulse response notch filter at 60 Hz with a quality factor of 10, and smoothed using a 200-point moving average window, to eliminate power-line interference and environmental noise. The heartbeat period of each signal was an average of more than 20 heartbeat cycles, sampled 10 seconds, 100 seconds, and 1000 seconds after recording, thus eliminating fluctuation over time.

## Results

**Figure 5:**
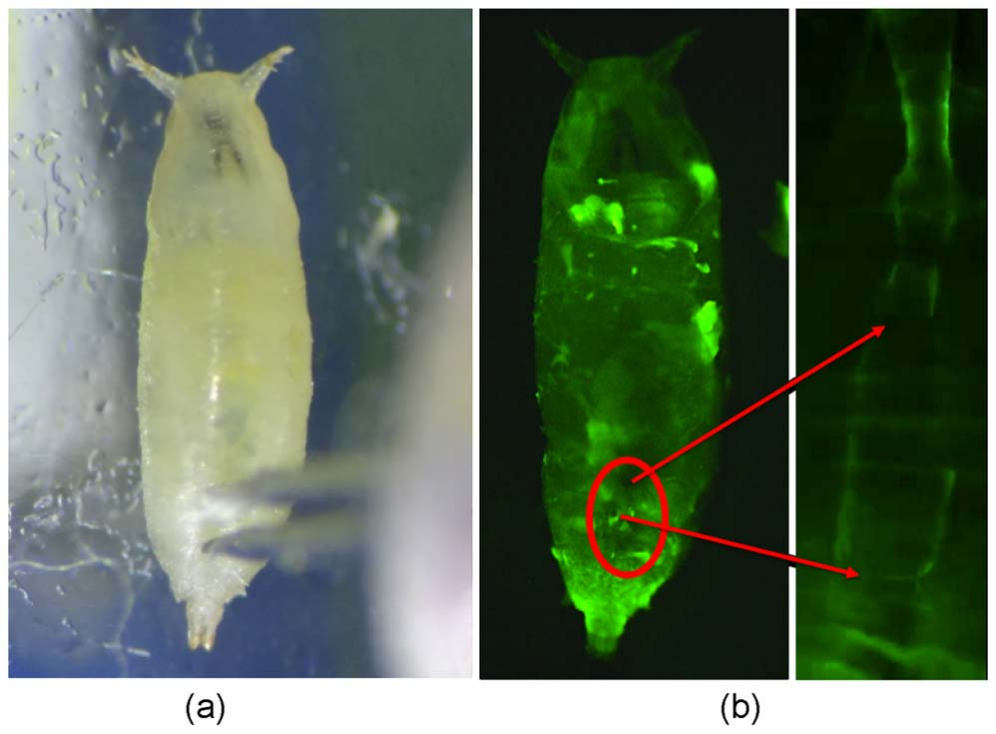
Photos of drosophila invasive ECG recording.

As shown in Figure 5(a), the electrodes used in the invasive method were inserted at A6–A8 of the pupa, as observed using a microscope. The depth of insertion was around 216 µm, which is the average of 5 insertions. Figure 5(b) shows a fluorescence photo of the pupa and its heart tube; the red circle denotes the locations at which the two probes were inserted. Insertion location and depth strongly affect ECG recording, and consistency was maintained in this study by using a probe holder, which was magnetically fixed on the probe station.

**Figure 6:**
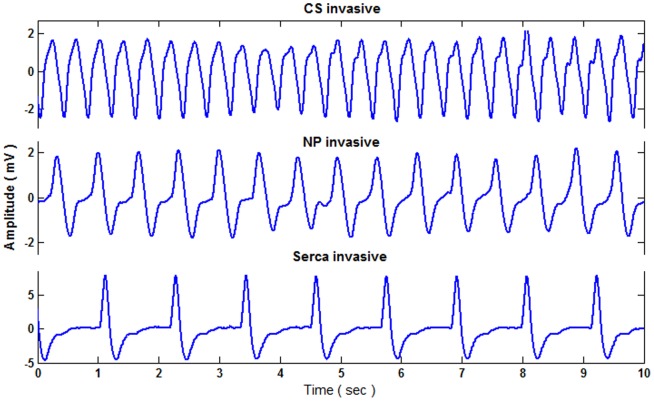
The ECG waveforms of drosophila invasively measured by insulated tungsten needle probe electrodes.

The ECG waveforms of the CS, NP, and SERCA-depleted phenotypes of *Drosophila*, recorded using the invasive method, are shown in Figure 6. As shown in the figure, differences in heart rates between the samples are obvious: CS exhibited the fastest heart rate, and SERCA-depleted exhibited the slowest. Moreover, NP exhibited a different HR than that of CS, thus emphasizing the impact on electrophysiological results caused by crossing with the fluorescent driver NP1029. Because conducting this electrophysiological experiment to record heart performance provided unaffected ECG results, the CS control measurements were more accurate than those obtained using the fluorescence based optical method [15] [16].

Invasive monitoring results in tissue injury and disturbance to the heartbeats of the animal under study. Recently, prior study has suggested that using optical systems disrupts heartbeats in flies, and causes the amplitude of heart rates to change substantially [17]. Furthermore, studies have shown that exposing the 3^rd^ larvae to light simultaneously promotes ventral muscle contraction and induces irregular heartbeats or cardiac arrest. However, noninvasive electrodes can be effectively used to record pupa heart beats [18]. The noninvasive method does not require destroying tissues or cells in the body of the animal under study, and is a convenient, fast, and accurate method for measuring heart rates. This finding is essential for studying the molecular mechanism involved in cardiac arrhythmia.

**Figure 7:**
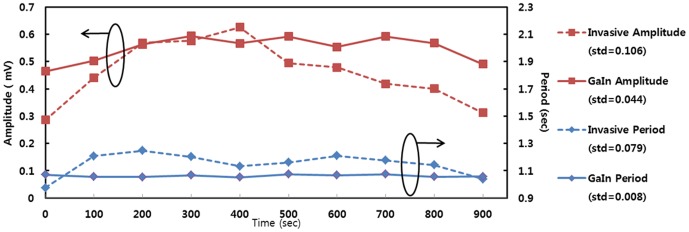
A 15 minutes heart performance of SERCA depleted group that is measured by two different methods. Solid line and dotted line present non-invasive and invasive recording, respectively.

Effective signal processing necessitates the stable, reliable recording of vital signs. Most electrophysiological experiments involving *Drosophila* ECG recording have entailed using dissection. However, dissecting the animal reduces the stability of its heart performance within several minutes. Moreover, because of heart-brain cross-talk, the destruction of organs may result in errors and changes in heart signals [6]. Figure 7 shows the heart performance of the SERCA-depleted specimen over 15 minutes, measured using two methods. The solid and dotted lines denote noninvasive and invasive recordings, respectively. The noninvasive method that involves using GaIn yielded superior stability both in amplitude and heart beat period. This result indicated that noninvasive ECG recording can be used effectively for heart performance recordings over an extended period.

## Discussion

**Figure 8:**
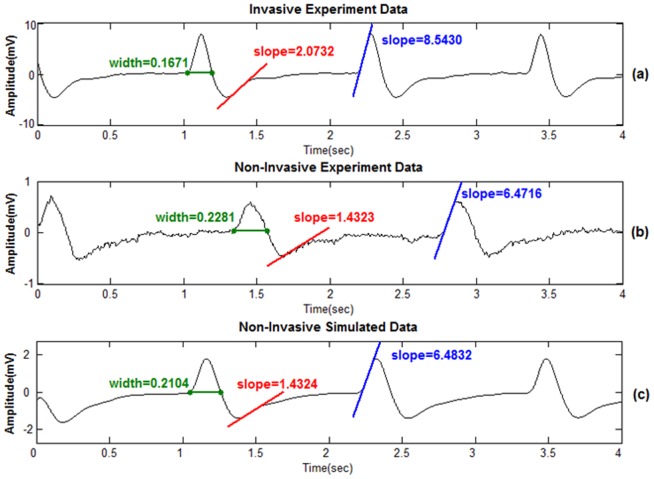
The ECG waveforms of Serca depleted group that (a) invasively measured by tungsten electrode (b) non-invasively measured by GaIn electrode (c) simulated with GaIn electrode.

Figure 8 shows the ECG waveforms of two SERCA-depleted specimens recorded using the invasive and noninvasive methods, respectively. As shown in the figure, the two waveforms are highly matched in beating periods and waveform features. To examine the feature differences between these two methods, the positive duration, depolarization slope, and polarization slope were calculated and marked on the waveforms. As shown in the circuit model established in Figure 1, the measured voltages of the invasive and noninvasive recording methods (*V_needel_* and *V_GaIn_*) were represented as (1) and (2):
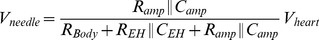
(1)

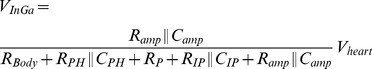
(2)


**Figure 9:**
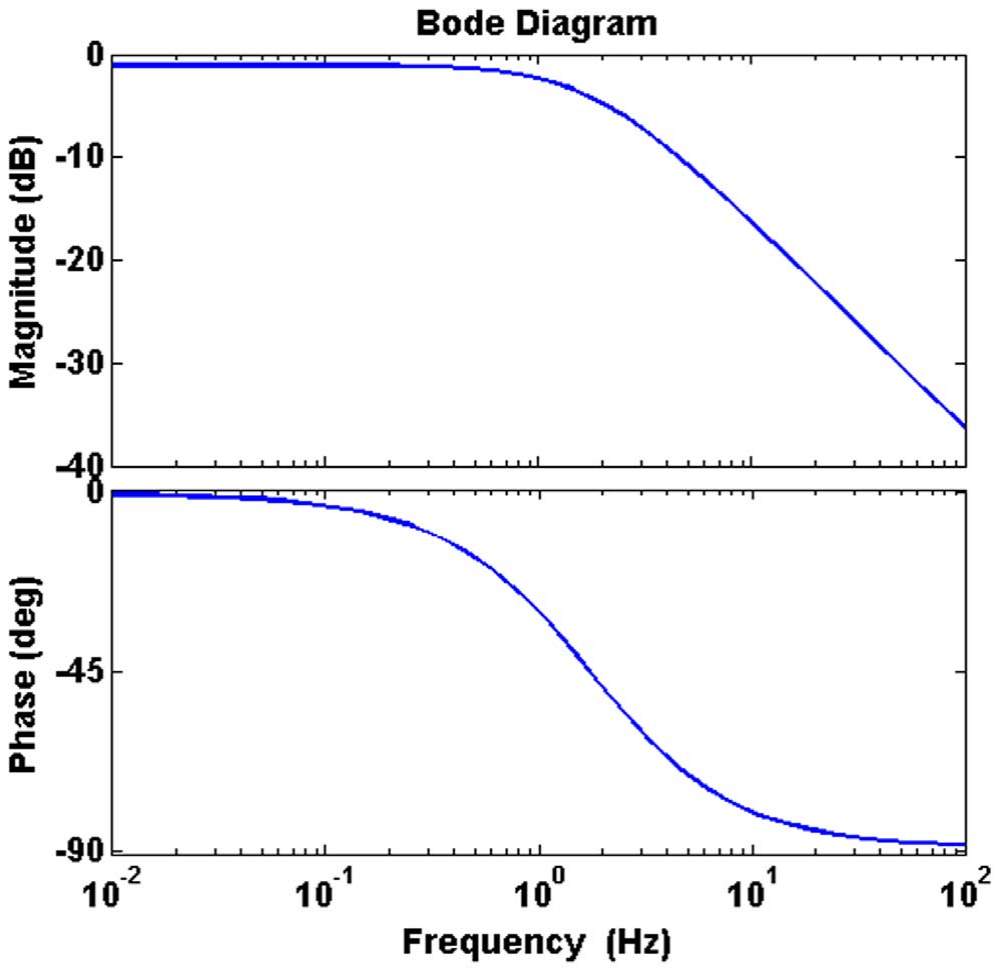
Bode diagram of response H(s).

Following (1) and (2), the features shown in Figures 8(a) and 8(b) were extracted, and the characteristics of R and C were modeled. The transfer function H(s) of *V_GaIn_*/*V_needel_* is expressed as (3):
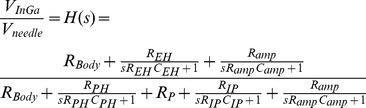
(3)in which H(s) results response of the low-pass filter. The Bode diagram of the transfer function is illustrated in Figure 9. For validating the accuracy of the circuit model, a simulated waveform of *V_GaIn_*, fitted by substituting *V_needle_* into (3), is plotted in Figure 8(c).

Both the measured and simulated analysis results of the noninvasive recording revealed that it had a slightly wider positive duration, and flatter polarization and depolarization slopes, compared with the invasive method. For noninvasive model, the parallel impedance R_IP_//C_IP_ causes slight low-pass filtering between *V_GaIn_* and *V_needle_*. However, the noninvasive measurement method still retains crucial ECG features. In cardiac arrhythmia research, this GaIn-based noninvasive measurement method can prolong the survival time of *Drosophila* specimens, and provide reliable ECGs for further signal processing.

**Figure 10:**
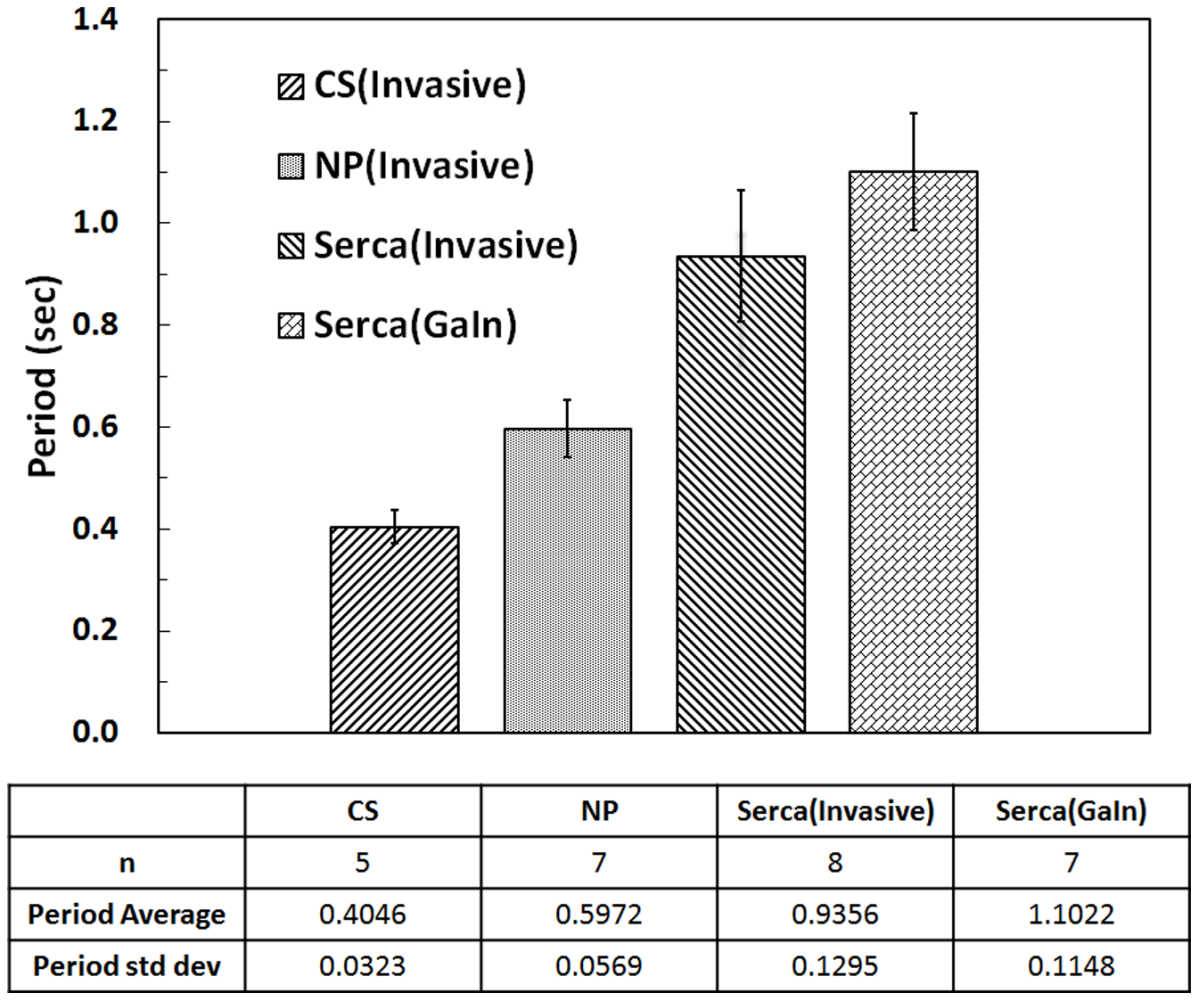
Summary of drosophila ECG Recording.

Figure 10 shows the n value and average and standard deviation of the heart beat period for each of the three *Drosophila* types studied using both recording methods.

The results indicated that the GaIn-based noninvasive recording method offered signal quality comparable to that obtained using invasive tungsten probes, while maintaining convenience, stability, and reliability.

## Conclusion

In this study, ECGs of *Drosophila* were recorded using both invasive tungsten needle probes and noninvasive GaIn electrodes. Noninvasive ECG recording by using GaIn electrodes was confirmed as a valid method for recording *Drosophila* pupae ECG signals within a limited contact area and signal strength. Thus, the observation of ECG changes in normal and SERCA-depleted *Drosophila* over an extended period is feasible. This method prolongs insect survival time while conserving major ECG features. This GaIn electrode system could provide a platform for electrophysiological signal research on the molecular mechanism involved in cardiac arrhythmia, as well as research related to drug screening and development.
